# Cortical Gamma Rhythms Modulate NMDAR-Mediated Spike Timing Dependent Plasticity in a Biophysical Model

**DOI:** 10.1371/journal.pcbi.1000602

**Published:** 2009-12-11

**Authors:** Shane Lee, Kamal Sen, Nancy Kopell

**Affiliations:** 1 Program in Neuroscience, Boston University, Boston, Massachusetts, United States of America; 2 Center for Biodynamics, Boston University, Boston, Massachusetts, United States of America; 3 Department of Biomedical Engineering, Boston University, Boston, Massachusetts, United States of America; 4 Hearing Research Center, Boston University, Boston, Massachusetts, United States of America; 5 Department of Mathematics and Statistics, Boston University, Boston, Massachusetts, United States of America; Université Paris Descartes, Centre National de la Recherche Scientifique, France

## Abstract

Spike timing dependent plasticity (STDP) has been observed experimentally *in vitro* and is a widely studied neural algorithm for synaptic modification. While the functional role of STDP has been investigated extensively, the effect of rhythms on the precise timing of STDP has not been characterized as well. We use a simplified biophysical model of a cortical network that generates pyramidal interneuronal gamma rhythms (PING). Plasticity via STDP is investigated at the excitatory pyramidal cell synapse from a gamma frequency (30–90 Hz) input independent of the network gamma rhythm. The input may represent a corticocortical or an information-specific thalamocortical connection. This synapse is mediated by N-methyl-D-aspartate receptor mediated (NMDAR) currents. For distinct network and input frequencies, the model shows robust frequency regimes of potentiation and depression, providing a mechanism by which responses to certain inputs can potentiate while responses to other inputs depress. For potentiating regimes, the model suggests an optimal amount and duration of plasticity that can occur, which depends on the time course for the decay of the postsynaptic NMDAR current. Prolonging the duration of the input beyond this optimal time results in depression. Inserting pauses in the input can increase the total potentiation. The optimal pause length corresponds to the decay time of the NMDAR current. Thus, STDP in this model provides a mechanism for potentiation and depression depending on input frequency and suggests that the slow NMDAR current decay helps to regulate the optimal amplitude and duration of the plasticity. The optimal pause length is comparable to the time scale of the negative phase of a modulatory theta rhythm, which may pause gamma rhythm spiking. Our pause results may suggest a novel role for this theta rhythm in plasticity. Finally, we discuss our results in the context of auditory thalamocortical plasticity.

## Author Summary

Rhythms are well studied phenomena in many animal species. Brain rhythms in the gamma frequency range (30–90 Hz) are thought to play a role in attention and memory. In this paper, we are interested in how cortical gamma rhythms interact with information specific inputs that also have a significant gamma frequency component. The results from our computational model show that plasticity associated with learning depends on the specific frequencies of the input and cortical gamma rhythms. The results show a mechanism by which both increases and decreases in the strength of the input connection can occur, depending on the specific frequency of the input. A current mediated by NMDA receptors may be responsible for the temporal course of the plasticity seen in these brain regions. We discuss the implications of our results for conditioning paradigms applied to auditory learning.

## Introduction

In many systems, synaptic plasticity is believed to depend on the spike timing of the pre- and postsynaptic cells [1],[2]. This spike timing dependent plasticity (STDP) has been observed *in vivo* [3] and has been studied in many *in vitro* preparations in the context of learning and sensory processing [1],[2],[4]. Though there have been many modeling papers on STDP and learning [5]–[8], the effect of rhythms on STDP has not been explored extensively, despite evidence that rhythmic activity is physiologically relevant. For example, a significant gamma rhythm (30–90 Hz) is present in cortical areas during attention to learning-related tasks [9] and is associated with the specificity of learning in auditory areas [10],[11] and with plasticity *in vitro* [12]. Spatially distinct gamma rhythms around 35 Hz are observed in granular Layer IV (LIV) and supragranular Layer II/III (LII/III) auditory cortical layers of monkeys that are awake but not involved in a task [13].

In this paper, we are concerned with an input that is periodic in the gamma frequency range, directed to an oscillatory network that produces an independent gamma frequency. The input is considered as an information encoding input, while the network is considered as a cortical network model in which gamma rhythms are modulated by non-specific drive (See Discussion). With temporal structure in the input and in the cortical network itself, the effects of even a simple STDP rule become much less transparent. If the input did not perturb the target output, the dynamically changing phase lags would be determined completely by the periods of the input and cortical network oscillation. When this is not true, as in the present work, the phase lags can change in a way that seems random. However, statistically there is a bias, and when the STDP effects of the different lags are averaged over some time window, cumulative changes in potentiation are seen. The frequency of the input influences the output network, and the phase difference between an input spike and a nearby output spike depends on the dynamics of the model system.

While N-methyl-D-aspartate receptor mediated (NMDAR) currents are known to be necessary in mediating many synapses affected by STDP [1],[2],[14], the precise effect on spike timing is less clear. We show that the slower decay kinetics of NMDAR currents in the excitatory cortical cell model affect the cycle of firing and increase the amplitude and time course of potentiation at that synapse. The plastic changes can be either potentiating or depressing, depending on the frequencies of the input and that of the receiving network. Indeed, we show that there are interspersed bands of input frequency over which there is potentiation or depression. Furthermore, a persistent stimulus can first lead to potentiation and then switch to depression. We explore the robustness of this system in detail.

Finally, we discuss a possible implication for our model results in the context of gamma rhythms nested in theta rhythms, and we discuss how the gamma frequency input in the model may represent an information specific corticocortical or thalamocortical input to a primary sensory cortical network with an independent gamma frequency.

## Results

**Figure 1:**
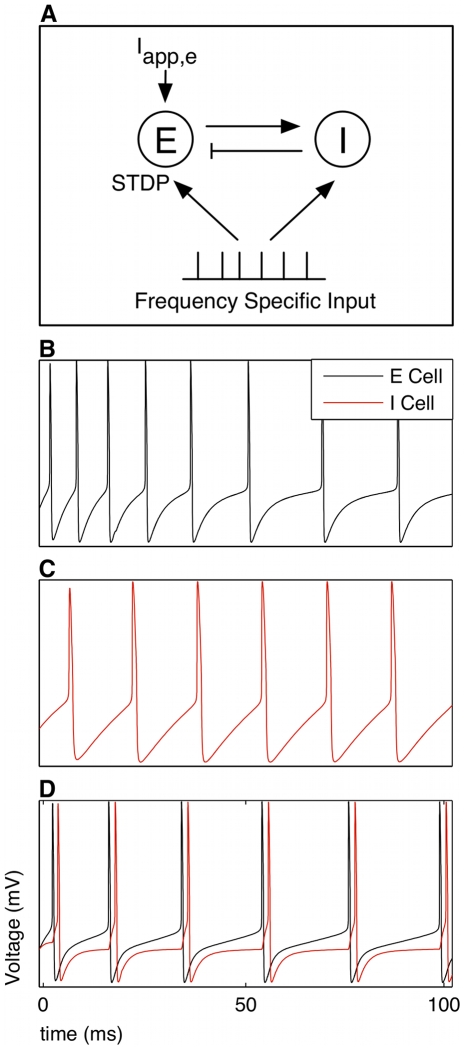
Network model generates gamma rhythms independently and receives gamma frequency input. (A) An excitatory regular spiking pyramidal cell (E) is mutually coupled with a fast spiking inhibitory basket cell (I). The E and I cells are single compartmental Hodgkin-Huxley type neurons. The E-I network is driven by 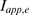 to spike at gamma frequencies. Both cells receive an input consisting of discrete spike times at gamma frequencies. Plasticity occurs only at the E cell synapse via STDP, manifested by changes in the AMPAR conductance. (B) The E cell is modeled after a regular spiking pyramidal cell in primary sensory cortex. It exhibits spike frequency adaptation when driven with an applied current. (C) The I cell is modeled after a fast spiking inhibitory basket cell that displays no spike frequency adaptation when driven with an applied current. (D) The coupled E-I oscillator can exhibit pyramidal-interneuronal gamma (PING) rhythms when the E cell is driven by an externally applied current. The I cell is not driven tonically in the simulations.

The model consisted of a conductance-based network model of primary sensory cortex that generated a gamma frequency rhythm, receiving an input of spike times that occurred at an independent gamma frequency (Fig. 1A). The network consisted of a single compartmental model of a regular spiking excitatory pyramidal cell (E), mutually coupled with a single compartmental model of a fast spiking inhibitory basket cell (I). Tonic drive 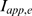 was applied to the E cell, generating pyramidal-interneuronal gamma (PING) rhythms [15] from 30–90 Hz (Fig. 1B–D). The E cell included dynamics modeled after glutamatergic NMDAR and *α*-amino-3-hydroxyl-5-methyl-4-isoxazole propionic acid receptor mediated (AMPAR) currents. The time constant for the decay of the NMDAR current 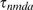 was varied in some simulations.

The input consisted of a list of discrete presynaptic spike time events with a period consistent with a gamma frequency (11–24 ms), independent of the network gamma rhythm. These spike time events activated glutamatergic responses in both the E and I cells in the model. Plasticity was modeled at the input E cell synapse by changes in the AMPAR conductance parameter 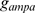 via a classical additive model of STDP using asymmetric parameters [5],[16]. Most simulations were run without noise in the input spiking, unless otherwise noted. In some simulations, a pause, or period of inactivity, was inserted in the input spiking. In other simulations, the time constant parameter for glutamate release 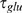 was varied. Multiple inputs were considered in certain simulations, for which a coherence parameter (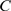) was defined. The mathematical details of the model are outlined in the Methods section.

For analysis, a frequency map was used to examine the global behavior of potentiation and depression over a wide range of input and network frequency regimes. In the map, frequency regimes were expressed as a ratio of the input frequency 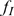 to the natural network frequency 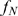, which was defined as the frequency at which the network oscillated in the absence of the input. This was necessary since the input itself affected the period of the network oscillation. The magnitude of potentiation in each frequency regime point was calculated by the area underneath the curve for the rise in 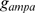 within a window of 100 ms centered around the peak value. For depressing frequency regimes, the magnitude of depression was calculated in a window centered on the lowest value of depression. Potentiating frequency regimes showed average increases in 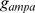 during this window. The mathematical details of the analysis are outlined in the Methods section.

### Broad frequency regimes of potentiation are input frequency dependent

**Figure 2:**
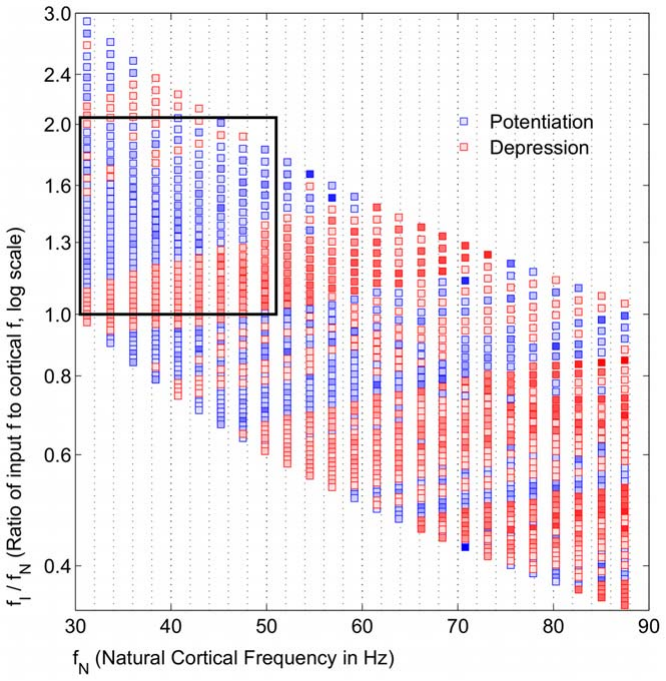
Frequency map shows broad bands of potentiation and depression that depend on 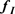. Distinct, broad bands of potentiation (blue) and depression (red) are interspersed for different natural network frequencies 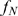 and input frequencies 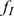. The color intensities are normalized to the maximum absolute values of either potentiation and depression, respectively. The values are averaged around a window 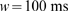 around the peak value. Each frequency regime was simulated without noise in the input spiking. The rectangle denotes the frequency space with the broadest band of potentiation, which we predict is likely a physiological range of input and cortical network frequencies.

The simplest case of a single gamma frequency input to the cortical network was considered initially. The model showed that broad bands of potentiation and depression existed for different frequency regimes that were periodic in the gamma frequency range (Fig. 2). Beta frequency (20–25 Hz) inputs exhibited bands analogous to the low gamma frequency region with the frequency ratio less than 1. For a constant applied drive to the E cell model, which set each 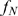, both potentiation and depression were possible, depending on the specific 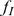. This suggested that the STDP mechanism naturally favored potentiating the responses of certain network frequencies to particular input frequencies. The inputs to the oscillator represented only two of several currents that affect the voltage dynamics of the network, and the resultant period of the network oscillation does not generally follow that of the gamma frequency input. With broad bands of potentiation, a range of input 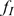 potentiated the E cell synapse being driven independently by 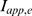. Narrower or absent bands of potentiation would have signified that a very precise input frequency would be required to potentiate the glutamatergic E cell synapse. This qualitative behavior also occurred with a constant NMDAR current having no decay (Data not shown), indicating that the global behavior of the model was more dependent on the frequency ratio than on the dynamics of the NMDAR current model, which played a more crucial role in regulating the specifics of the potentiation (See below).

**Figure 3:**
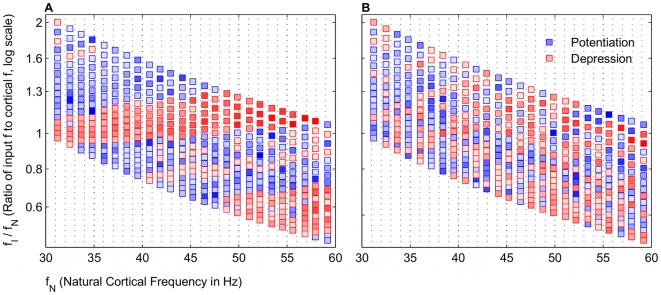
Frequency map structure requires gamma frequency input. Frequency ratio is plot as a function of 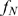. (A) Map is a subset of Fig. 3, shown for comparison with (B). With a gamma frequency input, broad bands of potentiation and depression are distinct. (B) With a Poisson distributed stochastic input, potentiation still occurs but not in regular bands.

Unlike the rhythmic gamma frequency inputs, Poisson distributed stochastic inputs did not lead to broad bands of potentiation (Fig. 3). However, when directed at the gamma generating network, theta frequency (4–12 Hz) input also did not exhibit these bands (Data not shown), though separate simulations, in which a pause was inserted into the input spiking (See below), may be thought of in terms of theta frequency modulation (See Discussion).

### Potentiation is transient and followed by depression

**Figure 4:**
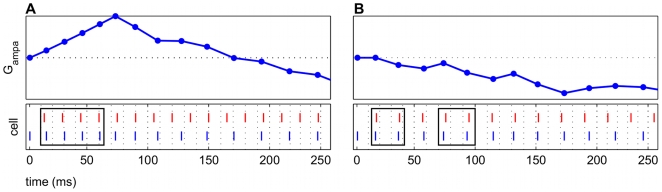
Potentiation and depression are expressed in the model as changes in the AMPAR conductance. The bottom panels show raster plots of the input spikes (red) and E cell spikes (blue). (A) Potentiation is shown for one frequency regime (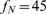 Hz, 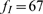 Hz). The boxes in the bottom panel indicate primary potentiating spike pairs in which the input spikes immediately precede the E cell spikes. (B) Depression is shown for one frequency regime (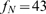 Hz, 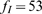 Hz). The boxes in the bottom panel highlight spike pairs that show E cell spikes (blue) preceding input spikes (red).

In potentiating frequency regimes, the rise of potentiation was generally followed by the subsequent decline. Therefore, each frequency regime had a maximal level of potentiation and a finite time over which potentiation lasted. Of the 483 potentiating frequency regimes shown in Fig. 2, 445 displayed this transition into depression within the 500 ms run. All 483 frequency regimes displayed the transition within 2000 ms. Potentiation occurred in the model when input spikes preceded E cell spikes and was strongest when the spike time difference was much less than the time constant for potentiation 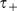 (Fig. 4A). Strong depression occurred when the E cell spiked before the presynaptic input (Fig. 4B).

**Figure 5:**
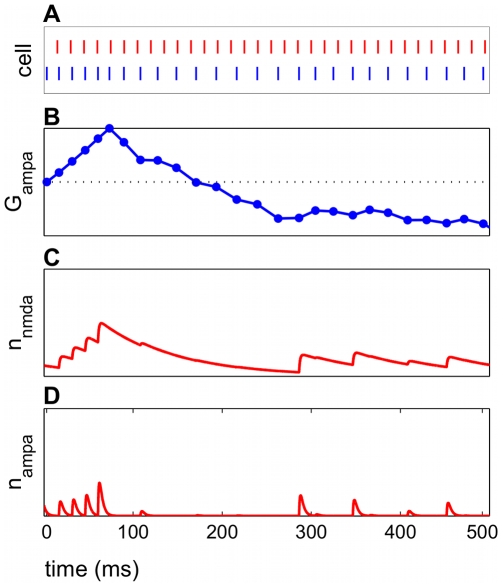
NMDAR currents exhibit a slower decay than AMPAR currents. A potentiating frequency regime is shown. (A) Raster plots for input spikes (red) and E cell spikes (blue) show positive spike pairs leading to potentiation. (B) AMPAR conductance over time shows potentiation and subsequent depression. (C) Open probability 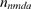 for 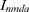 has local maxima when an input spike immediately precedes an E cell spike, signaling a glutamatergic current. The NMDAR current has a long decay time constant and therefore a prolonged effect on the E cell spiking. (D) Open probability 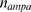 for 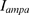 has a faster rate of decay than NMDAR events.

In a typical example of potentiation (Fig. 5), spike rasters confirmed positive spike pairs, in which the presynaptic input spike preceded the postsynaptic cortical E cell spike (Fig. 5A). The increase in 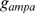 was also transiently observed (Fig. 5B). The model NMDAR current decayed more slowly than the model AMPAR current, demonstrated by the open probability kinetics (Fig. 5C and 5D). The slow decay of the NMDAR current dynamics had a prolonged effect on the cycle of E cell spike times, which affected the plasticity.

### Amplitude and duration of potentiation increased with larger 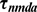


Though a constant instead of decaying activation of the NMDAR current model did not qualitatively change the frequency map, NMDAR currents in the model were a natural candidate for mediating the transition from potentiation to depression that occurred over time scales around 100 ms. Increases in 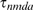 increased the average amplitude and duration of potentiation for a potentiating frequency regime.

**Figure 6:**
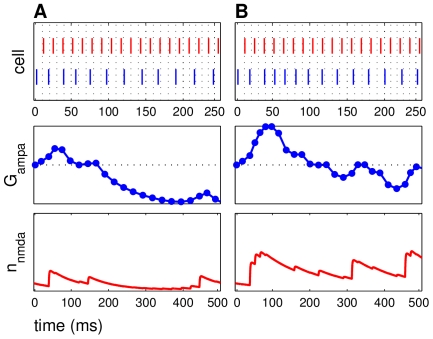
A longer NMDAR current decay results in larger average potentiation. The top panel in each subfigure shows a raster plot with input spikes (red) and E cell spikes (blue), enlarged over the first 250 ms to show detail. Second panels are AMPAR conductance over time. Bottom panels are NMDAR current open probabilities 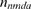. (A) The initial NMDAR current decay 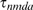 was 80 ms. This frequency regime (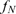 = 41 Hz, 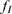 = 77 Hz) potentiated. Here, AMPAR and NMDAR events did not occur at regular intervals. (B) 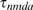 was increased to 130 ms. The same mean ISI and 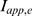 were used, corresponding to the same frequency regime as seen in (A). However, this frequency regime shows a greater amplitude of potentiation, seen in the rise in AMPAR conductance.

In the common frequency regimes that showed potentiation for a control 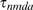 value of 80 ms and an increased 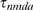 of 130 ms, there were increases in the amplitude of potentiation with the longer 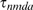 (Fig. 6), with a mean increase of 54%. Additionally, the time over which potentiation occurred was on average 2.9 times longer with the increased 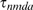. Decreases in 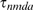 to 50 ms resulted in frequency regimes that continued to show potentiation compared to the control, but on average the amount of potentiation was 22% lower than the control (Data not shown).

The decay of the NMDAR current can account for the amplitude increase by prolonging E cell spiking activity. When an input spike immediately preceded an E cell spike, the STDP rule increased the AMPAR conductance, and the event-related NMDAR current was initiated. This raised conductance caused the next cycle of the E cell to fire faster, changing the rhythm of the network oscillator. However, as the NMDAR current decayed, the network rhythm was slowly pulled out of a potentiating frequency regime.

Due to the prolonged availability of slowly decaying NMDAR currents, a potentiating network rhythm could conceivably be prolonged by allowing for another positive spike timing event to occur. However, once the NMDAR current was extinguished completely, and the spiking rhythm fell out of a potentiating regime, only a strong positive spike timing event could have prolonged or re-initiated the potentiation. The long time course of the model NMDAR current may be an advantage of the system by allowing multiple gamma period spike timing events to occur during the NMDAR current decay. This sets a natural window for greater potentiation to occur, while also limiting the possibility for unlimited spiking pairing to occur after the initial potentiating event.

### Potentiation increases monotonically with constant NMDAR currents

**Figure 7:**
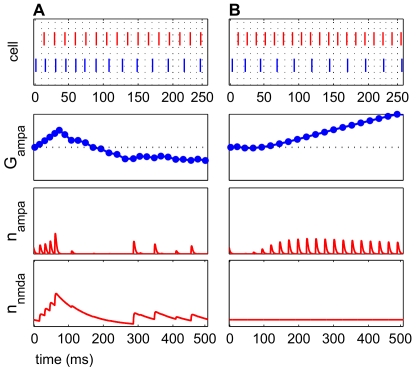
Potentiation increases monotonically with constant NMDAR currents with no decay. The top panel in each subfigure shows a raster plot, with input spikes (red) and E cell spikes (blue), enlarged over the first 250 ms to show detail. Second panels are AMPAR conductance over time. Third panels are AMPAR current open probabilities 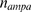. Bottom panels are NMDAR current open probabilities 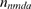. (A) A potentiating frequency regime (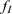 = 67 Hz, 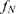 = 45 Hz) is shown with 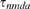 of 80 ms in which potentiation lasts only for a short time before becoming depression. (B) In this potentiating frequency regime (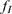 = 77 Hz, 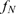 = 36 Hz) with a constant, non-decaying NMDAR current, potentiation increases monotonically. Glutamatergic events occurred on every cycle, since the spiking of the E cell was not being influenced by a slowly decaying NMDAR current. The regime used in (A) showed depression if the NMDAR current did not decay.

While transient potentiation was observed in nearly all simulations run with the standard 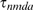 of 80 ms, monotonically increasing potentiation was observed in some simulations with a constant NMDAR current that exhibited no decay (Fig. 7).

The lack of NMDAR current decay dynamics eliminated the gradual change of the E cell spiking rate that is typically present. Since the rate of E cell spiking did not change dramatically without the NMDAR current decay, positive spike pairs were observed on each cycle of E cell spiking, contributing to a monotonic rise in potentiation.

### Pauses in input can prolong potentiation

Temporarily suspending presynaptic input prolonged potentiation in the time period after the pause. In all frequency regimes in which potentiation was observed without pauses, greater potentiation was observed after insertion of certain pause conditions, which were identified by start time and duration. Almost any length of pause resulted in post-pause potentiation, as long as spiking resumed before the NMDAR current was completely extinguished (approx. 80–130 ms). The model suggests that there was an optimal pause duration that resulted in maximal post-pause potentiation, which was dependent upon the specifics of the frequency regime.

**Figure 8:**
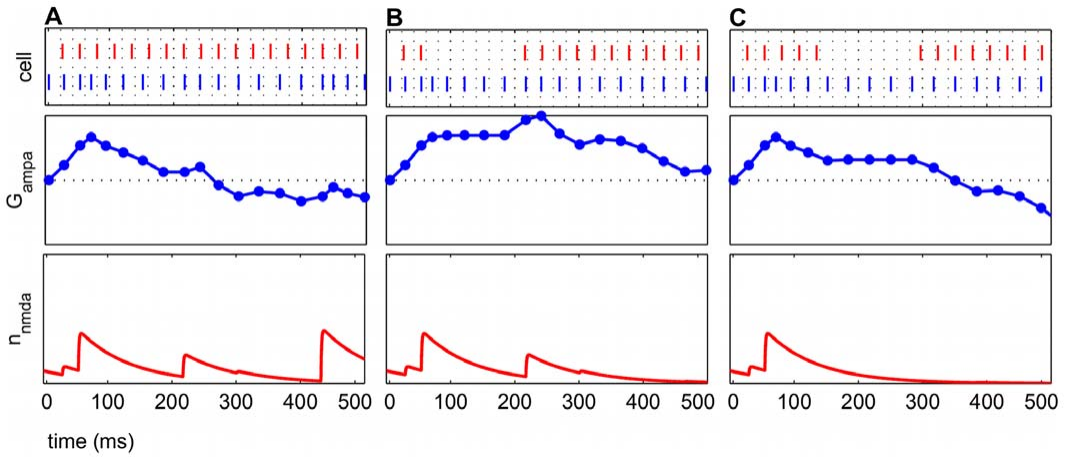
Inserting a pause in the input spiking prolonged potentiation. The pauses here are 125 ms long. The top panel in each subfigure shows a raster plot with input spikes (red) and cortical spikes (blue). Middle panels are the AMPAR conductance 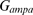. Bottom panels are open probabilities for NMDAR currents 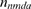. In each subfigure, the frequency regimes are the same (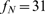 Hz, 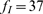 Hz). (A) The AMPAR conductance rises, signifying potentiation in the first 250 ms. (B) Inserting a pause of 125 ms in the input spiking (seen in the raster plot) suspended plasticity. The pause of this length begins during the height of potentiation (at approximately 75 ms), and potentiation resumed with the input. The long time course of the NMDAR current allowed the cortical spike rate to change gradually enough to keep the cortical network in a potentiating regime. (C) This pause is started at 150 ms and is the same length as (B). The pause ends beyond the natural decay of the NMDAR current, and post-pause potentiation did not occur, since the E cell spikes were no longer being sustained at a potentiating rate.

For all frequency regimes of potentiation, several positive spike pairs prolonged the potentiation. However, the increased activation drove the E cell spikes to precede the input spikes, and the potentiation turned to depression, as previously described. Further potentiation was observed (Fig. 8B) compared to the unpaused condition (Fig. 8A) when the pause in input spiking was 125 ms, less than 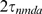. The AMPAR conductance was constant for the duration of the pause since no plasticity can occur, but once the input spiking was resumed, further potentiation occurred. When the input spiking was paused, starting at a time far after the peak of potentiation, depression persisted when the spiking resumed. This occurred because the NMDAR current had decayed completely, and no other positive spike pairs were observed (Fig. 8C).

### Increased coherence generally increases potentiation

In simulations with multiple inputs, the maximum contribution from each individual input was scaled inversely to the number of inputs. In this condition, coherence of inputs was necessary for strong potentiation. However, the simulations also showed that when inputs were slightly perturbed, potentiation was greater in some frequency regimes compared to fully coherent cases.

**Figure 9:**
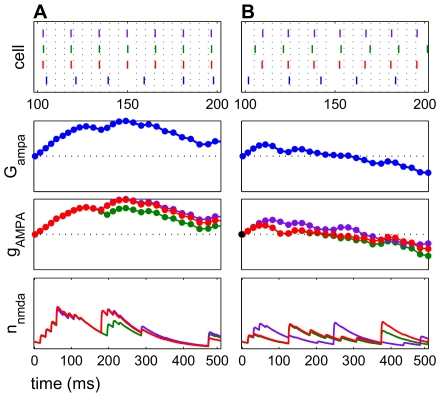
Greater coherence increases potentiation. The top panel in each subfigure shows a raster plot with spikes from each of 3 inputs (red, green, and purple) and spikes from the E cell (blue). The input spikes are randomly distributed with a variance of 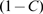 about the mean ISI. The time axis is enlarged to show detail. Second panels are total AMPAR conductance. Third panels are AMPAR conductance for each synapse for each individual input, rescaled. Bottom panels are NMDAR current open probabilities 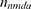 for each input. (A) C = 0.99 resulted in prolonged potentiation for this simulation with 3 inputs. (B) C = 0.10 resulted in diminished, brief potentiation, since input ISIs were still in a potentiating regime but not coherent.

For three inputs in a potentiating frequency regime, high coherence inputs (C = 0.99, Fig. 9A) exhibited greater potentiation than lower coherence inputs (C = 0.10, Fig. 9B). The applied current 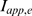, and therefore 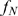, was identical for both cases. Because the inputs in the low coherence case arrived at drastically different times with respect to the E cell spikes, their individual effects were not strong enough to potentiate the E cell appreciably, as in the high coherence case.

**Figure 10:**
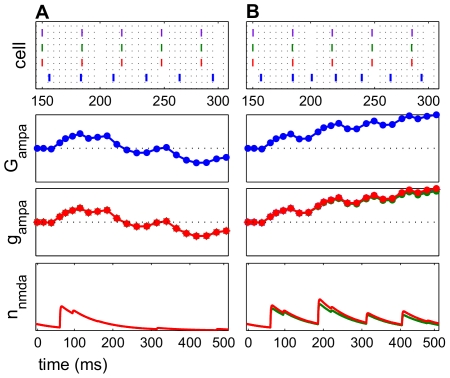
Noise can be beneficial for potentiation. The top panel in each subfigure shows a raster plot, with spikes of each of 3 inputs (red, green, and purple) and spikes from the E cell (blue). The time axis is enlarged to show detail. Second panels are total AMPAR conductance 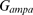 over time. Third panels show each individual 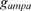 for each synapse, rescaled. Bottom panels are the NMDAR current open probabilities 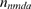. The frequency regimes are the same (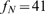 Hz, 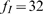 Hz. (A) 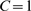. The inputs are completely regular, with no noise. The input ISI had a standard deviation (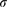) of 0. This frequency regime exhibited some potentiation, followed by the characteristic depression. (B) 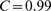, which corresponds to 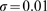. The average input ISI is preserved. The same frequency regime then exhibited prolonged potentiation, with no depression for the duration of this trial.

However, the difference between completely coherent inputs (C = 1, Fig. 10A) and slightly incoherent inputs (C = 0.99, Fig. 10B) exhibited the opposite behavior. For a frequency regime that showed potentiation when the inputs were completely coherent, slight decreases in coherence increased the potentiation. This was due to the increased robustness of the input spiking: with completely coherent inputs, as soon as the E cell spike preceded all of the input spikes, the entire system tended toward depression, as all of the spike pairs contributed to depression. However, less coherent inputs allowed for some spike pairs to contribute to potentiation while others contributed to depression. This small perturbation or noise in the input spiking may have allowed the potentiation to persist, since it did not require all of the inputs to spike before the E cell. Greater decreases in coherence, however, still led to depression, since far fewer positive spike pairs contributed strongly to an overall effect of potentiation.

### Prolonged glutamate exposure delays onset of peak potentiation

**Figure 11:**
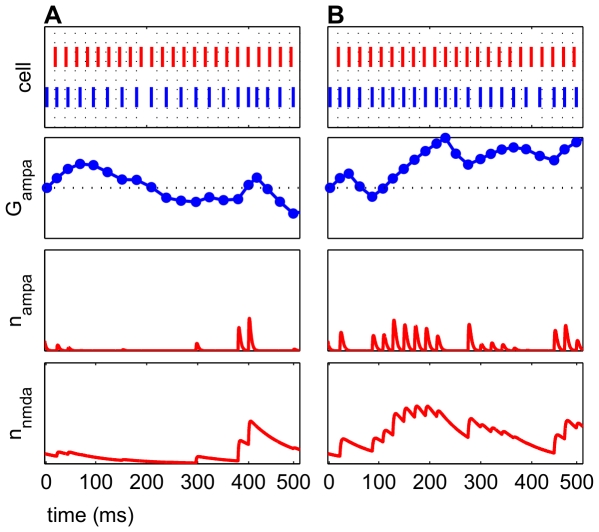
Greater glutamate availability increases potentiation. The top panel in each subfigure shows a raster plot of input spikes (red) and E cell spikes (blue). Second panels are AMPAR conductances 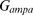 over time. Third panels are AMPAR current open probabilities 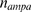. Bottom panels are NMDAR current open probabilities 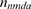. The frequency regimes are the same (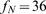 Hz. 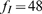 Hz). (A) 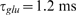. This frequency regime potentiated briefly. (B) 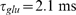. Input spike times are identical to (A), but greater and prolonged potentiation was seen.

**Figure 12:**
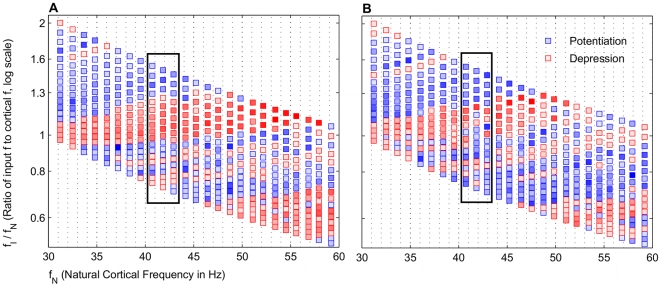
A larger decay constant for glutamate 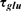 eliminates some bands in frequency map. Conductances are averaged around window 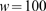 ms to calculate potentiation and depression. These simulations were run with noisy inputs (See Methods). (A) Frequency map for 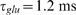. Standard bands of potentiation and depression were observed. (B) Frequency map for 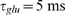. Several points of potentiation and depression were altered, and some 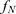 did not have broad bands of potentiation interspersed with depression, highlighted in boxed regions.

Changes in 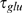 from 1.2 ms to 2.1 ms resulted in higher maximal potentiation and prolonged potentiation in some frequency regimes (Fig. 11). This result persisted when 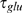 was modified to affect only AMPAR currents, only NMDAR currents, or both simultaneously. However, no gross qualitative changes were seen in the frequency map due to this change (Data not shown). In contrast, more dramatic changes to 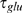 from 1.2 ms to 5.0 ms disturbed the frequency map, and the bands of potentiation and depression became less distinct (Fig. 12). At certain 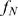, from 40–45 Hz, very little depression was seen irrespective of 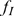, which suggested poor discrimination to the input frequency.

The longer 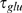 enabled more activation of the AMPAR and NMDAR currents in the model, since pre- and postsynaptic spikes could be paired within a longer interval. Persistent activation of glutamatergic conductances allowed for a greater contribution to the E cell's firing. In contrast, a shorter 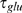 elicited fewer AMPAR and NMDAR currents, which enabled a faster frequency regime transition from potentiation to depression. To our knowledge, it is not known if glutamate is ever available for 5.0 ms endogenously, though bath application of glutamatergic agonists like kainate are common in slice preparations.

### STDP asymmetry is required for potentiation

**Figure 13:**
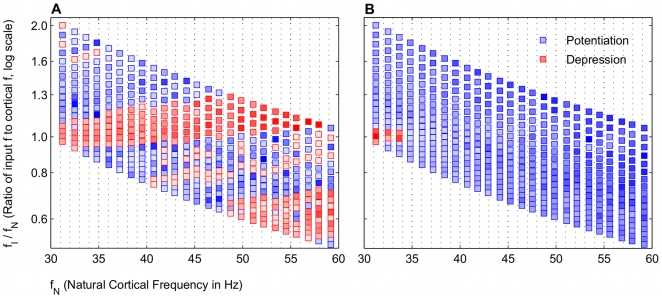
Frequency maps for symmetrical windows show nearly complete potentiation for all frequency regimes. Conductances are averaged around window 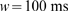 to calculate potentiation and depression. Potentiation is shown in blue, and depression is shown in red. (A) Temporally asymmetric windows. 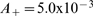, 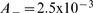, 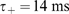, and 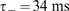. (B) Temporally symmetric windows, with the same amplitudes as (A) but with 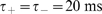. 443/450 shown frequency regimes potentiated in these conditions.

The output of the model depended heavily upon the STDP rule given by Eqn. (3). Both the temporal and amplitude asymmetries of the STDP rule were necessary for the frequency map structure seen in Fig. 2. Specifically, temporal asymmetry, in which 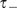 was greater than 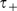, was required for bands of depression. There have been reports of temporal symmetry in some preparations [2],[17], though others appear to have observed temporally asymmetric windows [1],[7],[18]. The model results remain valid as long as 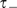 is greater than 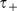. With temporally symmetrical windows, in which 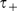 was equal to 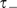 at 20 ms, every frequency regime became potentiating, irrespective of 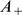 and 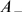 (Fig. 13). Further reductions of 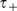 and 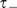 to 10 ms resulted in identical behavior (Data not shown). As expected, when 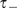 was less than 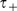, all frequency regimes potentiated as well, since the amount of time between spikes favored potentiation (Data not shown).

**Figure 14:**
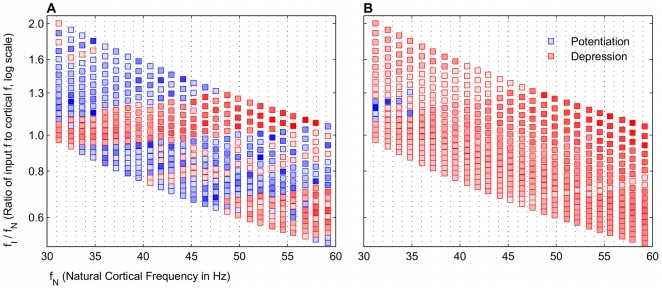
Frequency maps for 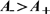 result in nearly complete depression. Conductances are averaged around window 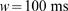 to calculate potentiation and depression. In both subfigures, 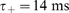 and 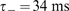. (A) Standard broad bands of potentiation existed when the amplitude for potentiation is greater than depression. 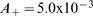, 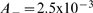. (B) 442/450 frequency regimes depressed when the amplitude for depression was greater than potentiation. 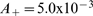 and 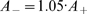.

Amplitude asymmetry, in which the ratio 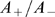 was greater than 1, was necessary for observing potentiation. When 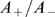 was 1 or slightly less than 1, favoring greater depression for the same 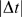, the frequency map no longer showed any bands of potentiation (Fig. 14). With this amplitude asymmetry, the maximal change in potentiation was greater than the maximal change in depression for the same 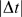 between pre- and postsynaptic spike times. This amplitude asymmetry was consistent with several experimental reports [1],[4],[17],[18]. Other models have also employed similar temporal and amplitude asymmetries [6],[19].

## Discussion

In this paper, for the first time, we investigate the consequences of STDP between two interacting gamma rhythms in a biophysical model. We show that potentiation and depression depend on the frequencies of both the input and the receiving network. The model suggests STDP as a mechanism by which responses to certain inputs can potentiate, while responses to other inputs depress. The bands of potentiation are broad with respect to input frequency and are robust to noise. The time and amplitude of the transient potentiation are primarily explained by phase precession that is dependent on the long time course of the dynamics of the NMDAR current in the model. The NMDAR current has long-lasting effects that persist beyond the initial activation of the receptors in the model, which is relevant to other biophysical models utilizing STDP, since these results suggest that STDP-mediated potentiation is a transient phenomenon when the input is prolonged. Additionally, the model suggests that pauses in the input spiking may increase the efficacy of the potentiation, which may have implications for tone shock presentations in an auditory fear conditioning paradigms. Finally, our network model could represent supragranular layer II/III of sensory cortex, and these results would apply to frequency-specific connections that may represent either a corticocortical input from granular layer IV or a direct thalamocortical input.

### Long NMDAR current decay and plasticity

The time course and amplitude of potentiation for a single frequency regime in the model increased with greater 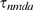. Longer decays are associated with earlier developmental stages [20],[21], at which time greater plasticity is exhibited [22]. Conversely, greater expression postnatally of the NMDAR subunit NR2A results in faster current decay [23]. The model results suggest that plasticity at synapses exhibiting shorter NMDAR current decays would be less likely to continue to potentiate with prolonged thalamocortical inputs.

### 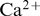 simplification in the model

The time scale over which the potentiation switched to depression in most frequency regimes (Fig. 5) was comparable to the decay time of the NMDAR current, motivating our focus on this current. However, the 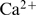 current dynamics also exhibit a time scale around 80 ms (Eqn. 1), but simulations that changed this time constant did not result in qualitative changes in either the frequency map or the average amplitude of potentiation (data not shown). However, it is thought that the 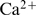-mediated component of NMDAR currents may be important in STDP [24],[25]. Other computational work addresses questions of how 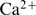 is used to induce plasticity [25]–[28]. Here, we focus on the implications of an STDP rule for plasticity rather than the underlying 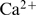-dependent mechanisms. For simplicity, we have not considered possible changes of 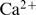 that are generated by NMDAR currents.

### Theta and gamma rhythms

While different gamma frequency inputs in the model led to broad bands of potentiation and depression (Fig. 2), theta frequency inputs did not. Yet gamma rhythms are often found nested within lower frequency theta rhythms [13],[29],[30], and functionally, theta frequency modulation of gamma frequencies has been implicated in short term memory performance [31]. Our network model is specifically a gamma generating model and does not explicitly take into account theta frequency modulation. As such, the lack of broad bands of potentiation by theta frequency spiking input is not necessarily surprising, since the input spike events did not occur frequently enough to sustain potentiation in a predictable manner. However, it may be possible to consider the effect of theta rhythm modulation of the network gamma within the context of our model. Our simulations showed that pauses can help to increase potentiation in certain cases. In our simulations, the optimal pause lengths (80–130 ms) approximately corresponded with the period of a theta frequency oscillation (80–250 ms, 4–12 Hz). The reduced excitability that marks the negative phase of the theta rhythm, approximately half of the theta rhythm period, may have a similar effect as the pauses in the input spiking. Future work could account for a theta frequency envelope that modulates the excitability of the gamma rhythm in the network model, which might result in further potentiation during the positive phase of the theta rhythm.

### Gamma rhythms in primary auditory cortex

In our simulations, a gamma frequency input was necessary for the presence of robust bands of potentiation in the frequency map, suggesting the involvement of a gamma frequency component in the input. Many aspects of our model parallel certain features of the auditory systems of some animals. The cortical network in the model most accurately depicts a supragranular gamma rhythm in primary auditory cortex (A1). However, there is uncertainty regarding the source of the input gamma rhythm. While it is known that there are tone frequency specific lemniscal inputs to A1 from the ventral portion of the medial geniculate complex of the thalamus (MGv) [32], the connections between granular and supragranular layers within A1 are less clear. As mentioned previously, at least one report has observed gamma rhythms that appear to be anatomically localized, with a supragranular gamma rhythm and a granular gamma rhythm [13]. Because the gamma frequency input in the model is simply a spike time list, and the learning rule is dependent only on spike timing, the input can represent either a granular input to the supragranular layer or a thalamocortical input directly to the supragranular layer. The granular gamma rhythm may be endogenous or be driven by activity of thalamic origin, suggested by anatomical evidence of a projection from the medial geniculate to Layers III/IV (LIII/IV) in A1 [33],[34]. It has been suggested that the MGv may convey temporally encoded information to the cortex [35], but it is not known whether or not there is a gamma frequency component in cells projecting from MGv during auditory tasks. In the visual system, there is evidence from the lateral geniculate nucleus of the thalamus suggesting that some thalamic cells generate gamma frequency oscillations that are coherent with corresponding cortical oscillations [36]–[38]. Further investigation will be necessary to understand the specifics of the thalamocortical and interlaminar anatomy and the roles of these possibly distinct gamma rhythms.

### Auditory thalamocortical plasticity

Certain predictions of our model may be testable in the thalamocortical auditory system of rats. In one circuit of thalamocortical auditory plasticity [32, Review], the specific thalamocortical input that carries tone frequency information ascends via at least two distinct pathways from the medial geniculate nucleus (MG). The lemniscal pathway terminates in LIII/IV of A1. The input to the cortical network in the model could represent this pathway. In some species, the non-lemniscal pathway terminates in both Layer I/II and LIII/IV of cortex from the medial portion of MG (MGm) [39],[40] and is thought to carry non-specific information [41], such as nociceptive information from a shock during a classical conditioning paradigm [32, Review]. Additionally, there are some suggestions that this pathway may carry both specific and non-specific information [32],[42]. In our model, the non-lemniscal pathway would be represented simply by the non-specific cortical drive (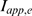). Each pyramidal cell in A1 responds selectively to a small range of tone frequencies, which peaks around the so-called best frequency (BF) [43]. In rats, this BF can shift to a new trained frequency (TF) in a classical conditioning paradigm [42, Review], which presumably requires some sort of synaptic modification. In these experiments, the neural response to the new TF is potentiated while the response to the old BF is slightly depressed.

The model results show that STDP may be able to account for this dual plasticity between the cortical gamma rhythm and the input gamma rhythm. The variable input frequency in the model represents the encoded TF and BF frequencies, which result in potentiation and depression, respectively, and is summarized in Fig. 2. The region highlighted in Fig. 2 shows that potentiation occurs most robustly when the gamma frequency of the encoded TF input is higher than the natural cortical frequency. We regard this as a prediction of our model.

Within the context of the auditory circuit, the model results suggest that long tone shock pair presentations in a fear conditioning paradigm can result in decreased efficacy of potentiation, especially if the presence of a slower rhythm to effectively pause the gamma rhythm is attenuated [44]. In many fear conditioning experiments, the tone and shock presentations on each trial co-terminate temporally [45]–[47]. Co-activation is represented in our model by simultaneously driving the cortical oscillator during the thalamocortical input. Potentiated frequency regimes all show potentiation followed by eventual depression (Fig. 5). This observation is related to the NMDAR current decay time and the availability of glutamate to the synapse. Applied to auditory fear conditioning, the model suggests that longer NMDAR current decays at the thalamocortical E cell synapse increase the maximal potentiation and prolong potentiation. Prolonging the tone-shock pair beyond 1–2 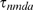, or 80–160 ms, would result in diminished potentiation or even depression. The model result that pauses can prolong potentiation may suggest that pulsed tone-shock presentations could result in faster, more robust potentiation. While pulsed tones have been employed in a few classical conditioning studies [48],[49], to our knowledge the effect of pulsed tones on the neural response, BF shift, or the efficacy of learning have not been systematically explored. Both the start time and the duration of the pause is significant in prolonging potentiation in the model (Fig. 8). The model results suggest that pauses effective in prolonging potentiation would start during the rise of potentiation and would persist for approximately the length of 1–2 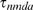. The intrinsic NMDAR current dynamics may need to be taken into consideration when choosing appropriate lengths for pauses. Further experimental work that assesses learning in an auditory fear conditioning experiment using pulsed tone shock pairs is needed to verify this result.

Our results suggest that the interaction of different gamma frequency rhythms can lead to potentiation or depression, depending on the frequency ratio. The amplitude and temporal profile of this potentiation is dependent on the NMDAR current dynamics, a result relevant to both models and experimental paradigms.

## Methods

### Frequency specific thalamocortical inputs

The frequency-specific thalamocortical input is modeled as a list of discrete spike time events with interspike intervals (ISIs) corresponding to rhythms in the gamma frequency range (11–24 ms). Results are reported without noise in the input ISI, unless otherwise noted. In noisy simulations, Gaussian random noise is applied with a mean equal to a predetermined ISI and a standard deviation of 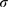 about the ISI. In most noisy simulations, 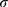 is 0.1.

Multiple inputs are also considered in certain simulations. Multiple inputs are modeled by distinct spike timing lists corresponding to each input, each with a specified average ISI and noise measure. Coherence 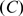 between the inputs is defined as 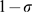.

The glutamate concentration 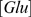 external to the cortical E and I cells is based on the timing of the most recent presynaptic event 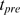. The amount of glutamate (Eqn. 1) decays exponentially from each synapse with a time constant 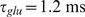 [50], unless otherwise noted.
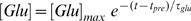
(1)


### Cortical network model

The single compartment E and I cells are Hodgkin-Huxley type cells that are coupled via synaptic currents and closely follow the formulation used in other work to represent a single auditory frequency channel [51]. Parameters for spiking currents follow previous models of a gamma rhythm [52]. The units for potentials are mV. All units for current are 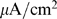. The units for conductance are 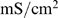. Opening rates (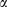) are expressed in 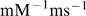 , and closing rates (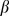) are in 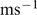. The specific membrane capacitance 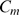 is 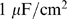. All alphabetical superscripts refer to particular gating variables or channel identifiers. The dynamics for the voltage of the E cell (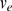) are given by:
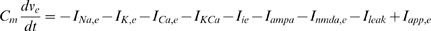
for 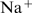 , 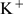 , 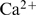 , 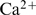-activated 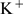 , a synaptic GABA*_A_*-like I to E, AMPAR, NMDAR, leak, and applied currents, respectively. The 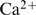 currents allow the E cell to exhibit spike frequency adaptation (Fig. 1B), as observed experimentally in neocortical pyramidal cells [3],[53],[54]. The strength of 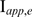 represents cholinergic or other neuromodulation [55],[56] to the Layer I apical dendrites of the pyramidal cells. In the absence of any plasticity, 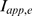 determines the natural firing frequency 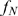 of the PING oscillation.

The voltage of the I cell (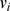) is given similarly to that of the E cell by:
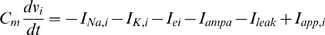
for 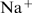 , 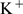 , a synaptic AMPAR-like E to I, AMPAR, leak, and applied currents. Because the I cell is driven by the E cell, 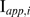 is 0. Alone, the I cell is a fast spiking cell that exhibits no spike frequency adaptation [54] (Fig. 1C).

The 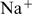 currents 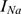 for both the E and I cell are given by:
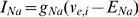


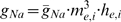


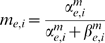


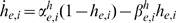


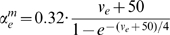


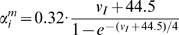


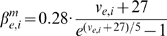
for constants 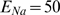 and 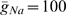.

The 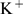 currents 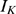 are given by:
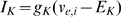


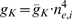


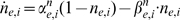


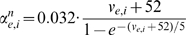


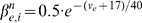
for constants 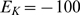 and 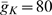.

The 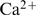 current 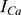 is given by:
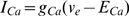


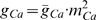


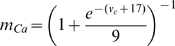
for constants 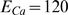 and 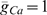.

The 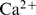-dependent 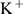 current 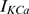 is given by:
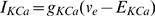


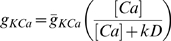


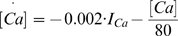
(2)for constants 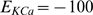, 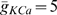, and 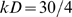.

The synaptic currents that couple the E and I cells are given by:
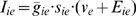


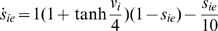
for constants 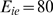 and 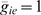, and
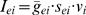


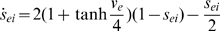
for constant 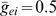. The leak current 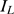 is given by:
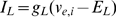
for 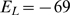 and 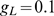.

The model includes glutamatergic AMPAR and NMDAR currents, which are both activated only within 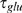 of the most recent thalamocortical spike time. For both the E and I cells, the AMPAR current 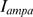 is given by:
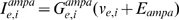


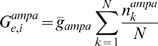


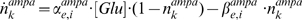
for the open probability 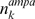, the number of presynaptic (thalamocortical) inputs 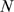, and the AMPAR current reversal potential 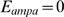. The opening and closing rate constants are 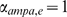, 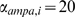, 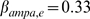, and 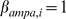.

The total AMPAR conductance 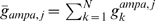 for 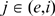 is a scalar value and is fixed for the I cell at 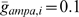. 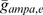 varies based on the spike timing rule. Each individual synapse begins with 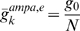 for a fixed initial value of the total conductance 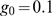 and the number of inputs 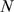. The total 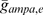 is therefore independent of 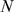 in this model.

For the E cell, the NMDAR current 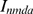 is given by [57]:
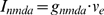


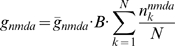


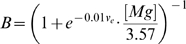


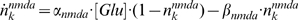
(3)


The constants are 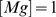 and 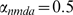. Unless otherwise noted, the closing rate 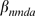 is 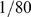, corresponding to an NMDAR current decay time constant of 80 ms.

In the absence of any presynaptic input, the spiking behavior of the mutually coupled E and I cells for a given 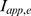 is shown in Fig. 1D.

### Thalamocortical plasticity

At the thalamocortical E cell synapse, the model utilizes a classical additive STDP rule that takes into account the contributions of every pair of pre- and postsynaptic spikes [5],[16]. Here, the presynaptic spike times are the thalamocortical input spike times, and the postsynaptic spike times are calculated when the E cell's voltage crosses an arbitrarily chosen +40 mV threshold. At each detection of an E cell spike, the model calculates the total contribution to the synaptic modification, which updates 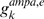 after detection of each postsynaptic spike and immediately affects the next cycle of firing.

The fractional synaptic modification 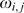 for each individual presynaptic (thalamocortical) spike time 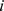 and each postsynaptic (cortical E cell) spike time 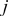 is calculated according to Eqn. (3) for 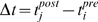 [5].
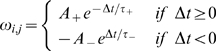
(4)Unless otherwise noted, the potentiation decays with a time constant of 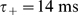, and depression decays with a time constant of 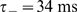 [5]. For a given positive spike pair, the maximal amplitude for modification 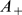 is 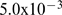. For a given negative spike pair, the maximal amplitude for depression 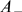 is 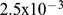.

The total synaptic modification 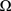 over all spike pairs is given by:
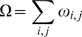
The AMPAR conductance for each input is given by the initial conductance 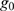 plus the synaptic modification:
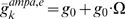
Additionally, at each synapse, the total maximal conductance 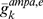 was restricted to 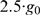, to ensure a maximal 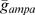 equal to 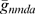, whose value was 0.25.

### Model simulations

Two primary parameters are tuned in each of the simulations: the current applied to the E cell (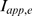) and the average interspike interval (ISI) of the thalamocortical input. Changing the 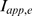 changes the natural frequency of the cortical oscillator 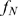. A range of 6–18 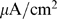 elicits a cortical oscillation within the gamma frequency range of interest (30–90 Hz). Changing the ISI from 11–33 ms changes the thalamocortical input gamma frequency 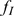 from 30.3 Hz to 90.9 Hz.

The closing rate for NMDAR currents 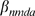 is equivalent to 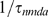 and is varied in some simulations. Other simulations investigate the effects of changing glutamate time constants for AMPAR and NMDAR currents, independently, motivated by evidence concerning the time courses of activation and decay [50],[58].

This model is based on several biophysical constraints and, though simplified, is not meant for mathematical analysis. Further work may elucidate some aspects of the results, including the potential resonance between the inputs and the network.

### Analysis

Potentiation and depression are defined in this paper by AMPAR conductance increases and decreases, respectively. The total potentiation 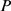 is defined by the area underneath the curve within a width 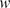 about the peak conductance value 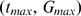, as shown in Eqn. (4 and 5). Depression is defined when 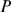 is less than zero.
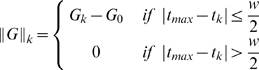
(5)

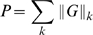
(6)


Each frequency regime is run for 500 ms of simulation time, in which both the thalamocortical input and cortical drive are activated. The simulations and subsequent analysis are run using MATLAB 7.6.0.324 (The MathWorks, Natick, MA). The differential equations are integrated using MATLAB's built in ode15s stiff solver of variable order and time step.
